# Amino Acid Substitutions in the C-Terminal Domains of Vip3Aa Enhance Insecticidal Toxicity

**DOI:** 10.3390/biology15171508

**Published:** 2026-09-03

**Authors:** Burcu Şahin, Patricia Hernández-Martínez, Juan Ferré

**Affiliations:** 1 Department of Biology, Faculty of Science, Muğla Sıtkı Koçman University, 48000 Muğla, Türkiye; bsahin@mu.edu.tr; 2 Department of Genetics, Institute of Biotechnology and Biomedicine (BIOTECMED), Universitat de València, 46100 Burjassot, Spain; patricia.hernandez@uv.es

**Keywords:** *Bacillus thuringiensis*, Vip3Aa90, site-directed mutagenesis, rational engineering, *Spodoptera littoralis*, *Spodoptera exigua*, *Grapholita molesta*

## Abstract

Vip3 proteins secreted by the entomopathogenic bacterium *Bacillus thuringiensis* (Bt) have an important role in biological control against economically important lepidopteran pests. The elucidation of Vip3 protein structures has helped to address the roles of domains and amino acid positions involved in toxicity, especially in the N-terminal domains I and II, thereby supporting their more efficient utilization. In this study, we evaluated the impact of combinations of critical amino acid substitutions, selected from previous studies, in domains IV and V of the Vip3Aa90 protein on its insecticidal activity against three lepidopteran pests. The double mutant S543N/I544L, triple mutants S543N/I544L/E627A and S543N/I544L/S686R, and quadruple mutant S543N/I544L/E627A/S686R were constructed in *Escherichia coli* by site-directed mutagenesis. Among these, only the Vip3Aa mutant proteins S543N/I544L/E627A and S543N/I544L/E627A/S686R could be expressed and purified for bioassays. Both mutant proteins had similar toxicity against *Spodoptera littoralis*, showing higher insecticidal activity than the wild-type (WT) Vip3Aa90 at the LC_90_ level. However, at the LC_50_ level, only a slight improvement in toxicity was observed for the quadruple mutant. In the case of *S. exigua*, no significant difference in toxicity was observed for either of the two mutant proteins with respect to the WT at either LC level. Interestingly, for *G. molesta*, though the toxicity of the triple mutant did not differ significantly compared to that of the WT protein, that of the quadruple mutant showed a marked decrease in toxicity of over 10-fold. This study revealed that combining selected amino acid substitutions in domains IV and V can enhance Vip3Aa90 toxicity against some lepidopteran species but can be either neutral or even deleterious in others.

## Simple Summary

*Bacillus thuringiensis* Vip3 proteins are useful in biological control because of their specificity and low cross-resistance with Cry proteins. Therefore, it is important to reveal the insecticidal activities of these proteins by enhancing their efficacy. In this study, we aimed to investigate the insecticidal activity of specific amino acid alterations in domains IV and V of the Vip3Aa90 protein. Four mutant constructs were generated by site-directed mutagenesis, but only two mutant proteins could be expressed, purified, and tested against three lepidopteran pests from the genera *Spodoptera* and *Grapholita*. Bioactivity assays showed that triple and quadruple mutants increased toxicity against *Spodoptera littoralis*. However, no increased insecticidal activity was observed for *Spodoptera exigua* and *Grapholita molesta*. The findings suggest that the effect of amino acid substitutions in the C-terminus of Vip3Aa is species-specific and, in some cases, may significantly increase toxicity against some lepidopteran species.

## 1. Introduction

*Bacillus thuringiensis* (Bt) is a ubiquitous sporulated bacterium with the capability of surviving and colonizing various niches. The entomopathogenic properties of some isolates make this bacterium a successful biopesticide source. The target-specific and environmentally friendly insecticidal proteins produced by this bacterium have been widely used for many years in the control of various agricultural pests through Bt-based formulations or the development of Bt transgenic crops [1]. The main commercial insecticidal proteins of Bt have been Cry and Cyt proteins, which have been the most studied and whose mechanisms have been well elucidated [2]. However, a significant number of pests exhibit low or no sensitivity to Cry proteins, and the emergence of resistance to these proteins has been widely reported [3]. Vips (vegetative insecticidal proteins), another important Bt insecticidal family, are referred to as second-generation insecticidal proteins and have emerged as a promising strategy for the management of resistant pest populations [4]. They bind to different binding sites in the insect midgut membrane and do not exhibit cross-resistance with Cry proteins, which increases their potential applicability [5]. Among Vips, Vip3 proteins, together with Cry proteins, are incorporated into a pyramiding strategy to control pests that have developed resistance in crops [6].

The *vip3* gene, which consists of approximately 790 amino acids, codes for an 89 kDa protein with a conserved N-terminus and a variable C-terminus. In recent years, studies on structural analyzes and the insecticidal mechanism of Vip3 have gained momentum [4,7,8,9,10]. Cryo-EM and structural analysis revealed that Vip3A consists of five domains [11]. While the N-terminal domains (I–II) are essential for maintaining the tetrameric structure, the C-terminal domains (III–V) are thought to be involved in receptor binding for DIII and to contain glycan-binding motifs for Domains IV and V [10]. Domains IV and V are predicted to function as carbohydrate-binding modules (CBMs). They can bind to different target cells via their distinct putative glycan-binding pockets [8]. Furthermore, in recent years, it has been shown that Domain V contributes to insecticidal activity by binding to the peritrophic matrix [7]. Therefore, it is worthwhile to investigate whether these domains have an effect on toxicity.

Several gene mutation experiments targeting the N-terminus and C-terminus were conducted to investigate the insecticidal mechanism of Vip3A proteins [6,12,13,14,15]. The C-terminal region of Vip3A proteins is critical for both stability and insecticidal activity. The mutation, even a single amino acid modification in this region, can lead to significant changes in its insecticidal activity against different insect species [5]. In addition to single mutations, double and triple amino acid changes have also been tested to determine whether they have an effect that enhances the insecticidal activity. For instance, Yang et al. [16] tested double S543N/I544L, triple S543N/I544L/E627A, and S543N/I544L/S686R Vip3Aa mutants against different lepidopteran larvae. While single and double mutants were not more toxic than WT Vip3Aa, S543N/I544L/E627A and S543N/I544L/S686R triple mutant Vip3Aas showed 7.3-fold and 2.8-fold higher insecticidal activity against *S. frugiperda*, respectively. S543N/I544L/S686R mutant Vip3Aa also exhibited 3.2-fold toxicity against *H. armigera*.

This study aimed to test whether combinations of previously described substitutions in domains IV and V of Vip3Aa could also improve toxicity against two *Spodoptera* species and *G. molesta*. Triple S543N/I544L/E627A and quadruple S543N/I544L/E627A/S686R mutant proteins of Vip3Aa90 were obtained for bioassays. While both mutant proteins showed higher insecticidal activity at the LC_90_ level against *S. littoralis* than the wild-type protein, the quadruple mutant demonstrated significantly higher toxicity at the LC_50_ level.

For the first time in this study, the quadruple mutant was generated, and the two combinations of mutant Vip3A proteins were tested against *S. littoralis*, *S. exigua*, and *G. molesta*.

## 2. Materials and Methods

### 2.1. Site-Directed Mutagenesis of Domains IV and V of Vip3Aa90

*Escherichia coli* BL21 cells carrying the *vip3Aa90* gene (NCBI accession XFO99222) in the pet14b vector were inoculated onto LB-ampicillin (50 μg/mL) agar plates and incubated overnight at 37 °C. A single colony was inoculated into 10 mL of LB-ampicillin (50 μg/mL) medium and incubated overnight at 37 °C with shaking at 200 rpm. Plasmid DNA was isolated with the Nucleospin Plasmid kit (Macherey-Nagel, Düren, Germany). The concentration of nucleic acid was measured by the NanoDrop 2000 (Thermo Fisher Scientific, Wilmington, DE, USA).

There are four mutants of Vip3Aa90: S543N/I544L, S543N/I544L/E627A, S543N/I544L/S686R, and S543N/I544L/E627A/S686R. They were constructed by site-directed mutagenesis using the QuickChange protocol. PCR amplification was performed with three pairs of primers shown in Table 1. For the E627A forward primer, the primer sequence used by Yang et al. [16] was chosen. The other forward primers were designed with modifications. Reverse primers and primers for sequencing of mutant PCR products were also designed in this study (Table 1). The PCR mixture included 0.3 mM dNTP mix, 5 µL 5× PCR buffer, 0.5 U Kapa HiFi DNA polymerase (Roche, Basel, Switzerland), 0.3 µM of forward and reverse primers, and 20 ng DNA template in a total volume of 25 µL. The PCR protocol consisted of an initial 3 min denaturation at 95 °C followed by 16 cycles with 20 s denaturation at 98 °C, 30 s of annealing at 58 °C, 52 °C, 57 °C for S543N/I544L, S543N/I544L/E627A and S543N/I544L/S686R, respectively, and 7 min of extension at 72 °C. A final extension step of 15 min at 72 °C was added. The digestion of PCR products with DpnI (New England Biolabs, Ipswich, MA, USA) was performed as described by Banyuls et al. [15].

Transformation of the plasmid containing the *vip3Aa* mutant gene into *E. coli* DH10B competent cells was performed using heat shock [17]. After transformation, colonies were picked and inoculated onto LB-ampicillin agar plates, followed by incubation at 37 °C overnight. Selected colonies were then inoculated into 4 mL of LB-ampicillin medium and incubated at 37 °C, 190 rpm, overnight. Following this, plasmid DNA from these colonies was isolated and used for sequence analysis with the primers in Table 1 to confirm the mutation sites. First, the mutant S543N/I544L plasmid of Vip3Aa90 was constructed. After confirming the mutation sites by sequencing, the S543N/I544L/E627A and S543N/I544L/S686R mutant constructs were generated using the S543N/I544L plasmid as the parental template. The quadruple mutant S543N/I544L/E627A/S686R was subsequently constructed using the S543N/I544L/S686R plasmid as the template for mutagenesis (Supplementary Figure S1).

### 2.2. Expression and Purification of Mutant Vip3Aa90 Proteins

After confirming mutant plasmids by sequencing, they were transformed into BL21(DE3) *E. coli* cells for expression. The protein expression was performed as described by Şahin et al. [18] with some modifications. Accordingly, a single colony of recombinant *E*. *coli* BL21(DE3) cells containing wild-type and mutant *vip3Aa90* genes was inoculated and grown in 20 mL of LB-ampicillin (50 μg/mL) medium overnight at 37 °C with shaking (200 rpm). The precultures were transferred to 700 mL of LB-ampicillin (50 μg/mL) medium and incubated at 37 °C, 180 rpm. When the OD_600_ reached 0.6–0.9, the cultures were induced with 1 mM isopropyl β-D-1-thiogalactopyranoside (IPTG). Following overnight incubation at 37 °C, 200 rpm, the cells were collected by centrifugation at 8800× *g* for 30 min at 4 °C. The pellets were resuspended in 20 mM phosphate buffer (pH 7.4) containing NaCl (0.5 M), lysozyme (3 mg/mL), DNAse (10 μg/mL), and phenylmethylsulfonyl fluoride (PMSF) (100 μM), and incubated with shaking at 37 °C for 30 min. Samples were sonicated twice for 60 s with a 10 s interval, followed by centrifugation at 15,000 rpm for 30 min; the obtained supernatants were then filtered through a 0.22 μm cellulose acetate filter. The lysates were checked by SDS-PAGE analysis before purification.

The wild-type and mutant Vip3Aa90 proteins were purified using HisTrap FF crude Lysate columns (Cytiva, Uppsala, Sweden) as previously described by Şahin et al. [18]. The concentration of purified fractions was measured at 280 nm using a Nanodrop spectrophotometer. Fractions containing the highest concentrations of Vip3Aa were combined and dialyzed overnight against 20 mM Tris buffer pH 8.6, 150 mM NaCl, and 5 mM EDTA. The protein samples were run on an SDS-PAGE gel, and protein concentration was determined by densitometry analysis before bioassays (Supplementary Figures S3–S5).

### 2.3. Bioassays with Wild-Type and Mutant Vip3Aa90 Proteins

The quantitative surface contamination assays were performed against *S. littoralis*, *S. exigua*, and *G. molesta* neonate larvae to test the insecticidal activity of mutant Vip3Aa90 in the protoxin form. Six to seven serial dilutions (0.24–180 ng/cm^2^ for *S. littoralis*, 0.11–85 ng/cm^2^ for *S. exigua*, and 1.6–405 ng/cm^2^ for *G. molesta*) (Supplementary Tables S1–S3) were used to conduct dose–response assays. A total of 50 µL of each dilution of protein was added onto artificial diet [19] in each 2 cm^2^ multiwells. Sixteen neonate larvae were used per concentration. The protein storage buffer was used as a negative control. Bioassays were placed in a bioclimatic chamber and incubated at 25 °C, 60 +/− 10% RH, 16:8 L/D conditions. The assays were conducted in triplicate, except for the triple mutant protein in *S. exigua* and the WT for *G. molesta*, which were performed in duplicate. The replicates were performed on different days, using different batches of Vip3Aas. Mortality was checked after 7 days and evaluated as functional mortality, which included dead larvae and those that remained at L1 without the ability to continue their normal development. The Polo-PC program (LeOra Software, Berkeley, CA, USA, 1987) was used for statistical analyses. The fiducial limits of LC_50_ and LC_90_ values were considered to determine a significant difference between assays.

## 3. Results

### 3.1. Expression and Purification of Vip3Aa90 and Mutant Proteins

**Figure 1:**
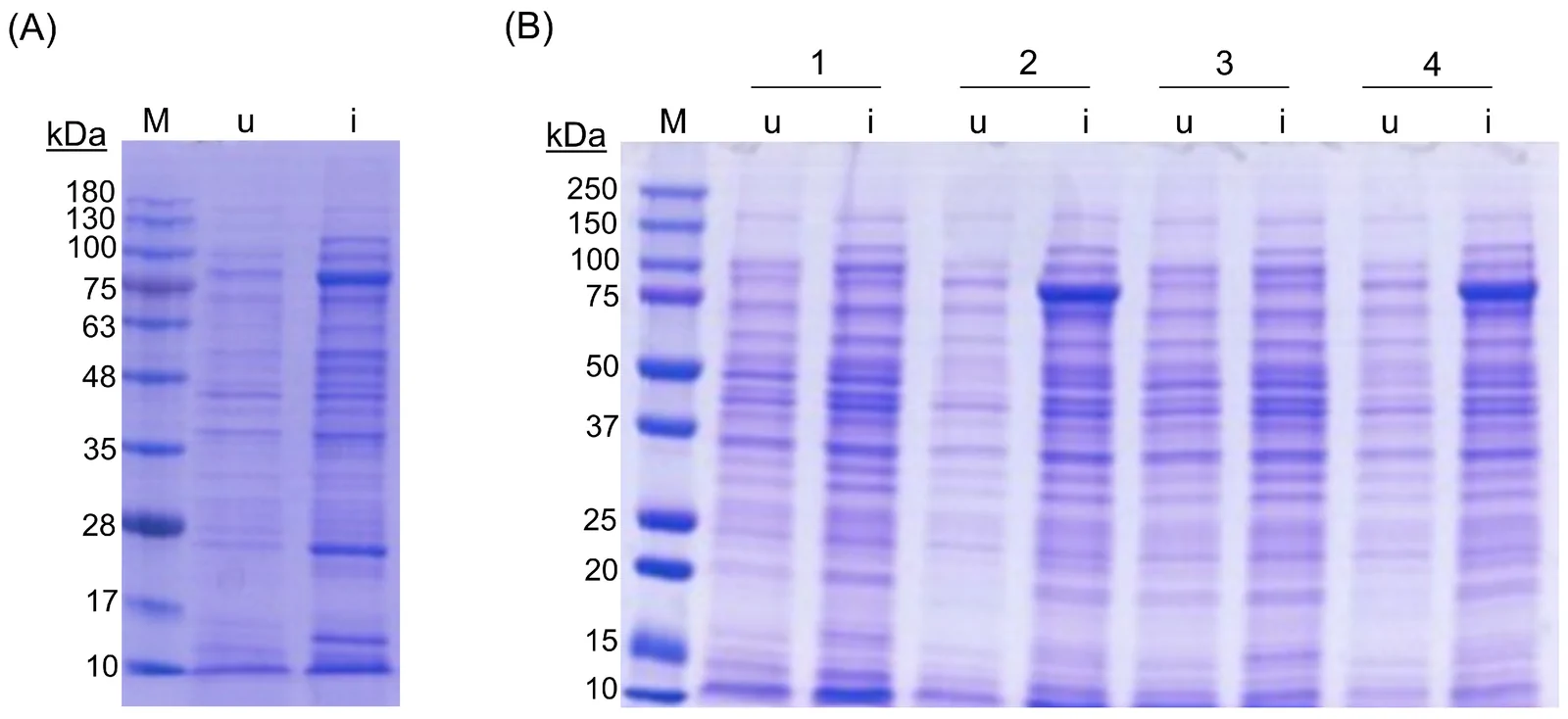
SDS-PAGE gel for expression of Vip3Aa90 (~89 kDa). (**A**) Induction of wild-type Vip3Aa90. (**B**) Induction of mutant Vip3Aa90s, 1: S543N/I544L (uninduced), 2: S543N/I544L/E627A (induced), 3: S543N/I544L/S686R (uninduced), 4: S543N/I544L/E627A/S686R (induced), M: molecular weight marker; u: uninduced cultures, i: induced cultures with IPTG.

Wild-type Vip3Aa90 and its mutants S543N/I544L/E627A and S543N/I544L/E627A/S686R were successfully expressed after induction with IPTG (Figure 1). In contrast, the double mutant (S543N/I544L) and the triple mutant (S543N/I544L/S686R) could not be successfully expressed under the conditions used (Figure 1), although these mutant plasmid constructs were successfully generated (Supplementary Figure S1).

**Figure 2:**
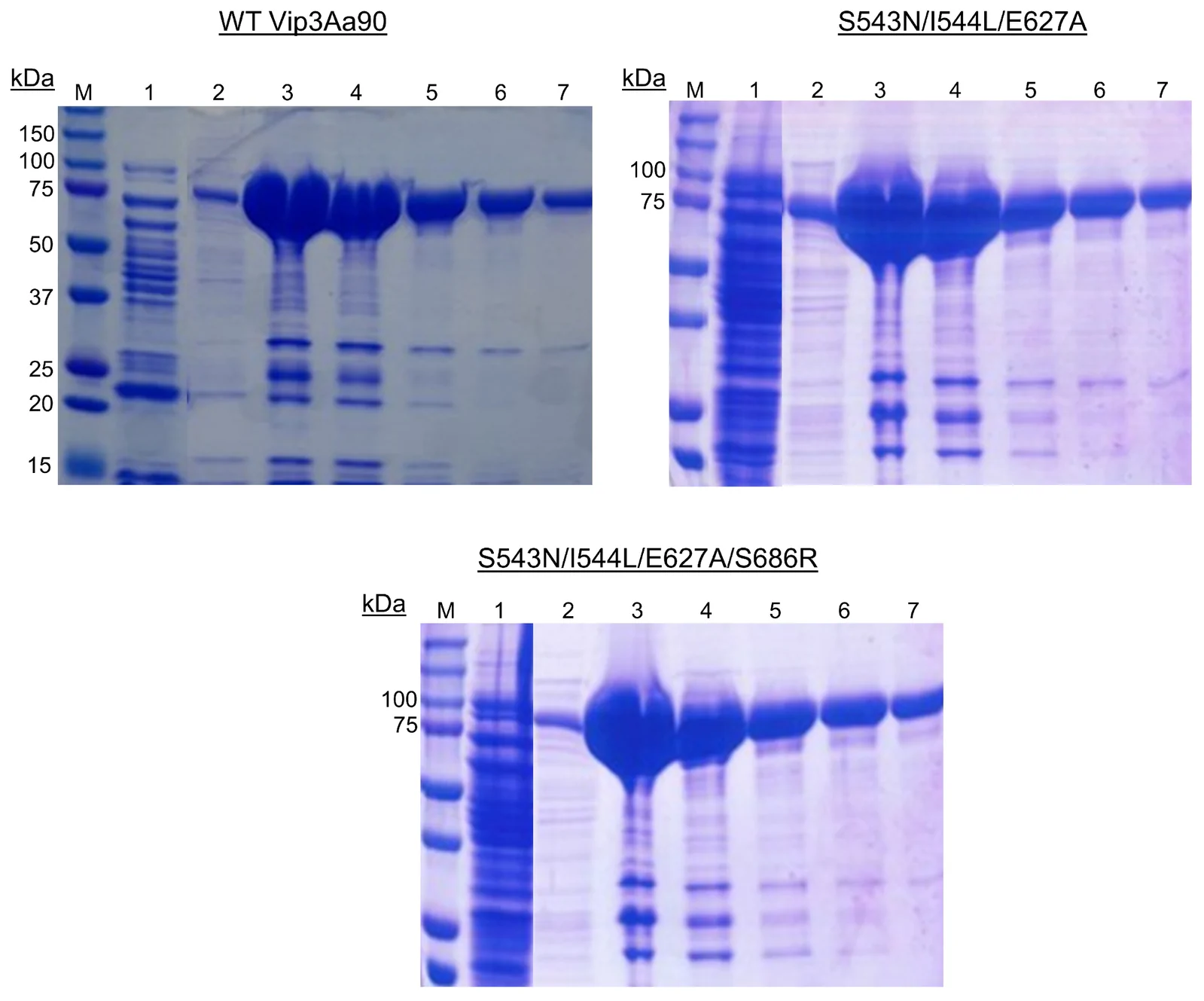
Purification of WT and mutant S543N/I544L/E627A and S543N/I544L/E627A/S686R Vip3Aa90 by affinity chromatography using His-Trap FF columns. M: Molecular weight marker, 1: lysate, 2: first elution fraction, 3–7: elution fractions to be combined for assays.

Expressed proteins were purified by affinity chromatography, and the purity was checked by SDS-PAGE analysis (Figure 2, Supplementary Figure S2).

### 3.2. Insecticidal Activity of Vip3Aa90, and Its Mutant Proteins

The insecticidal activity of the expressed and purified proteins (protoxins) was tested against three different lepidopteran species at different concentrations (Table 2). LC values with non-overlapping fiducial limits were considered to be significantly different. Bioassay results with *S. littoralis* showed that both the triple (S543N/I544L/E627A) and quadruple (S543N/I544L/E627A/S686R) mutant proteins were significantly more toxic than the WT protein at the LC_90_ level, with 11.6- and 15.6-fold lower values, respectively. However, at the LC_50_ level, just a slight improvement in toxicity was observed for the quadruple mutant. In the case of *S. exigua*, no significant difference in toxicity was observed for either of the two mutant proteins with respect to the WT at either LC level. Interestingly, for *G. molesta*, though the toxicity of the triple mutant did not differ significantly compared to that of the WT protein, that of the quadruple mutant showed a marked decrease in toxicity of over 10-fold.

## 4. Discussion

In insect resistance management (IRM), Vip3s have offered a solution to the development of resistance to currently used Bt-based biopesticides or Bt transgenic plants, as they provide an alternative way to kill the insect pests. Furthermore, the deployment of improved Vip3 variants may contribute to improved efficacy of pest control in field conditions. For instance, modified Vip3C, found to be toxic against *S. frugiperda* in both laboratory and field experiments, was shown to have different binding sites than Cry proteins, and this transgenic maize provided protection against Cry1F-resistant pests [20]. Transgenic corn expressing the T686R mutant Vip3Af protein, with significantly higher insecticidal activity against some of the pests, showed high levels of resistance against most pests [21]. Therefore, obtaining different mutation combinations of Vip3 and testing them on different species could benefit this strategy.

**Figure 3:**
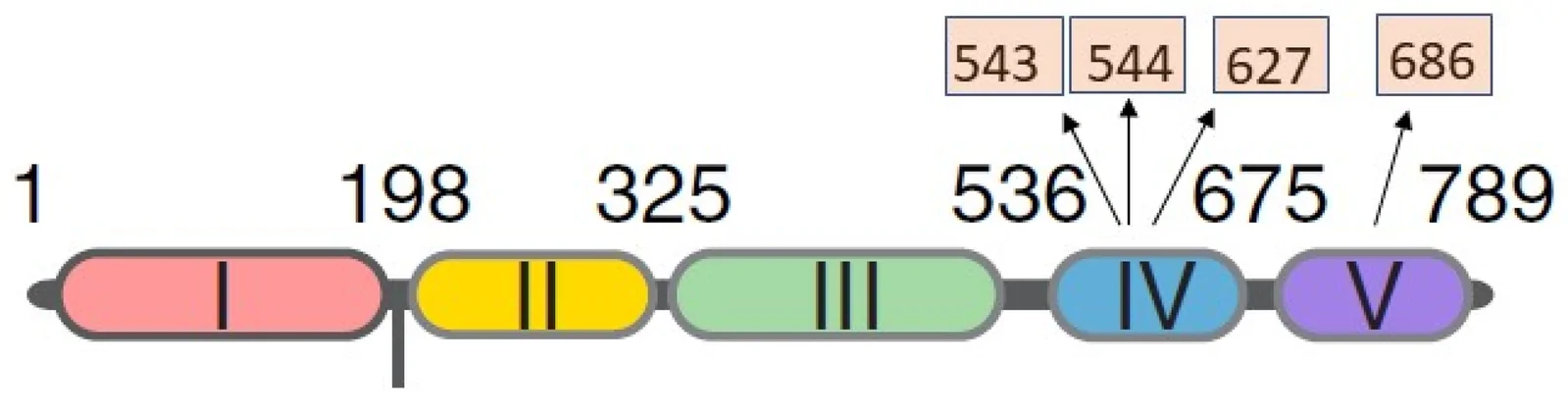
A schematic diagram showing the five domains of the Vip3A protein (color-coded) in which the related residues and substitutions (indicated with arrows) are located (adapted from Núñez-Ramírez et al. [11]).

The substitution residues used in this study, S543, I544, and E627, are located within domain IV (from 537 to 667), whereas S686 is positioned in domain V (from 679 to 789) [8]. Figure 3 shows the domains in which they are located within the scheme presented by Núñez-Ramírez et al. [11]. The combinations of mutations were selected from those that have shown high insecticidal activity in previous studies and are suggested to be critical in toxicity. A study by Chi et al. [22] has shown that S543N, I544L, and S686R mutant Vip3Aa11 exhibited significantly increased insecticidal activity against *S. exigua* compared with that of the wild-type (WT) protein. In the study conducted by Chi et al. [23], quantitative bioassay results showed that none of the Vip3Aa11 mutants, E627A, Y619A, and W552A proteins, tested against *S. exigua* and *Helicoverpa armigera*, were significantly more toxic; in fact, some of them significantly reduced the toxicity. In another study, the T686R mutant Vip3Af showed significantly increased toxicity to *S. exigua*, *Ostrinia furnacalis*, and *Mythimna separata* larvae compared to the WT Vip3Af protein [21].

Yang et al. [16] already showed enhanced activity of triple mutants S543N/I544L/E627A and S543N/I544L/S686R against *S. frugiperda* and *H. armigera*. In this study, our main contribution was testing the same constellation of substitutions with a new quadruple combination, S543N/I544L/E627A/S686R, in Vip3Aa90 against *S. littoralis*, *S. exigua*, and *G. molesta*. The triple and quadruple mutants tested in this study showed significantly higher insecticidal activity compared to wild-type Vip3Aa90 at the LC_90_ level against *S. littoralis*, but were not more toxic against *S. exigua* and *G. molesta* (Table 2). LC_90_ point estimates suggest that the two mutant proteins exhibit higher activity than WT. However, due to the wide fiducial limits associated with WT LC_90_ estimation, the fold differences should not be considered as exact effect sizes and should be interpreted cautiously as approximations. Previously, the same mutant was tested against various lepidopteran pests and found to be significantly highly toxic to *S. frugiperda* [16].

We could not test the S543N/I544L and S543N/I544L/S686R mutant proteins in bioassays because their protein expression failed. It is important to note that, since these two constructs could not be evaluated, the contribution of each individual substitution cannot be fully determined for the insect species tested in the present study. Furthermore, potential combined effects among these substitutions cannot be ruled out. Although the same mutants had been previously expressed in *E. coli* [16], the failure in the present study may be due to expression- or construct-dependent reasons such as differences in protein stability, folding, or expression efficiency [24,25]. Inducing protein expression in *E. coli* with IPTG at 37 °C may have caused the cells to rapidly synthesize proteins, leading to misfolding and low soluble protein. In addition, this condition may have contributed to some proteolytic/aggregation-derived low molecular weight products in expressed and purified proteins (Figure 2) [26].

In this study, a new construct (quadruple mutant S543N/I544L/E627A/S686R) was generated to evaluate whether it exhibited significantly greater toxicity than the wild-type Vip3Aa90 or the triple mutant. Substitution of the neutral serine (S) with the basic arginine (R) at position 686 has been reported to enhance proteolytic activation of the protein under the alkaline conditions of the midgut of lepidoptera [22]. Our results indicate that quadruple mutant S543N/I544L/E627A/S686R does not confer an advantage in terms of insecticidal activity compared to triple mutant S543N/I544L/E627A. Although the quadruple mutant displayed significantly higher toxicity than wild-type Vip3Aa90 against *S. littoralis*, it showed a markedly reduced insecticidal activity against *G. molesta* (Table 2). Furthermore, no significant difference in insecticidal activity was observed between triple and quadruple mutants against *S. littoralis*. In *S. exigua*, the triple mutant was found to be more toxic than the quadruple mutant at the LC_50_ level. In the case of *G. molesta*, the triple mutant was found to be more toxic than the quadruple mutant at both LC_50_ and LC_90_ levels. Our findings suggested that the S543N/I544L/E627A/S686R quadruple mutant Vip3Aa90 did not confer an advantage in terms of insecticidal activity compared to S543N/I544L/E627A. This may be due to the structural or conformational changes that negatively affect protein efficiency or stability; however, further structural analysis is needed to strengthen this hypothesis.

Similarly, in previous studies, we observed that *Spodoptera* and *Grapholita* species responded differently to the same Vip3A protoxins (Table 2, Supplementary Tables S1–S3). The difference in susceptibility to the Vip3A protoxin among species may be due to species-specific receptors or differences in other steps in the mode of action of Vip3Aa [10]. These interspecific differences highlight the need for further research to elucidate their underlying causes. Understanding these mechanisms will be crucial in determining the specific contribution of these residues to protein function and to enhance the rational design of next-generation Vip3 variants.

## 5. Conclusions

In this study, we constructed multiple mutations in Domains IV and V of Vip3Aa90 and revealed the insecticidal activity of the triple S543N/I544L/E627A and quadruple S543N/I544L/E627A/S686R mutants against agricultural lepidopteran pests. The results demonstrated that the effect of these changes is species-specific. Compared to the WT protein, the S543N/I544L/E627A and S543N/I544L/E627A/S686R mutant proteins increased toxicity in *S. littoralis*, though they did not show any significant improvement against *S. exigua* and *G. molesta.* The results, based on laboratory bioassays using purified protoxins, indicate that changes in specific residues may have a different effect on toxicity depending on the insect species targeted.

## Figures and Tables

**Table 1:** Primers used for mutagenesis of *vip3Aa90* and sequencing of mutant PCR products.

Procedure	Mutation	Primer	Sequence (5′−3′)	Codon (aa) (wt/Mutation)
Mutagenesis	E627A	fwd	GAAGATACAAATAATAATTTA**GCT**GATTATCAAACTATTAATAAACG	GAA (E)/ GCT (A)
		rev	GTTTGATAATC**AGC**TAAATTATTATTTGTATCTTCATAATG	
	S686R	fwd	CAAATAATTGGACG**CGT**ACGGGATCAACTAATATTAGC	AGT (S)/CGT (R)
		rev	GTTGATCCCGT**ACG**CGTCCAATTATTTG	
	S543N/I544L	fwd	TATTAGCAATATTGTAGAGAACGGG**AACTTA**GAAGAGGACAATTTAGAGCCGTGG	TCC (S)/AAC (N) ATA (I)/TTA (L)
		rev	CTAAATTGTCCTCTTC**TAAGTT**CCCGTTCTCTACAATATTGCTAATA	
Sequencing	E627A and S686R	fwd	GTAGATCATACAGGCGGAGT	-
		rev	GTACAATAGGACCACCATATAAATTATTCC	-
	S543N/I544L	fwd	TAAGATATGAGGTAACAGCGAAT	-
		rev	CCCGTACTCGTCCAATTAT	-

The sites of mutagenesis were underlined and in bold case.

**Table 2:** Toxicity of Vip3Aa90 protoxin and its mutant proteins against lepidopteran species.

Insect	Vip3Aa90 Protoxin	Slope ± SE	LC_50_ (ng/cm^2^) (95% FL)	LC_90_ (ng/cm^2^) (95% FL)	χ^2^ Values	Heterogeneity
*S. littoralis*	1	1.3 ± 0.15	12.68 (4.42–34.10)	122.78 (44.17–665.56)	29.145	2.0818
	2	2.45 ± 0.33	3.14 (1.97–4.73)	10.53 (6.91–18.18)	6.8044	0.76
	3	2.72 ± 0.38	2.65 (1.65–4.02)	7.87 (5.15–13.56)	7.6108	0.59
*S. exigua*	1	1.76 ± 0.30	4.10 (1.99–7.90)	21.95 (11.0- 61.00)	8.5471	0.78
	2	1.99 ± 0.23	2.01 (1.40–2.84)	8.82 (5.73–17.37)	16.699	1.2845
	3	2.82 ± 0.35	6.33 (4.47–9.06)	18.03 (12.26–30.64)	10.2186	0.93
*G. molesta*	1	1.73 ± 0.25	11.21 (7.09–17.84)	61.9 (33.6–216)	11.767	1.6810
	2	1.67 ± 0.24	4.72 (2.18–9.02)	27.68 (14.02–77.91)	13.186	1.1987
	3	1.54 ± 0.19	120.40 (72.23–210.26)	811.56 (425.25–2063.13)	8.1982	0.75

1: WT, 2: S543N/I544L/E627A, 3: S543N/I544L/E627A/S686R.

## Data Availability

All data presented in this study are included in the article and Supplementary Material.

## Supplementary Materials

The following supporting information can be downloaded at https://www.mdpi.com/article/10.3390/biology15171508/s1. Figure S1: Sequence analysis of mutant Vip3Aa90 constructs; Figure S2. Purification of WT and mutant S543N/I544L/E627A and S543N/I544L/E627A/S686R Vip3Aa90 proteins by affinity chromatography using His-Trap FF columns; Figure S3. SDS-PAGE gel for densitometry analysis of WT Vip3Aa90; Figure S4. SDS-PAGE gel for densitometry analysis of Vip3Aa90- S543N/I544L/E627A; Figure S5. SDS-PAGE gel for densitometry analysis of Vip3Aa90- S543N/I544L/E627A/S686R. Table S1. The concentrations of Vip3Aa90 WT and mutant protoxins and numbers of dead larvae for each replicate against *S. littoralis*; Table S2. The concentrations of Vip3Aa90 WT and mutant protoxins and numbers of dead larvae for each replicate against *S. exigua;* Table S3. The concentrations of Vip3Aa90 WT and mutant protoxins and numbers of dead larvae for each replicate against *G. molesta.*

## Abbreviations

The following abbreviations are used in this manuscript:

Bt	*Bacillus thuringiensis*
WT	Wild type
Vip	Vegetative insecticidal protein
IPTG	β-D-1-thiogalactopyranoside
PMSF	Phenylmethylsulfonyl fluoride
IRM	Insect resistance management
CBMs	Carbohydrate-binding modules

## References

[1] Kumar P., Kamle M., Borah R., Mahato D.K., Sharma B. (2021). *Bacillus thuringiensis* as microbial biopesticide: Uses and application for sustainable agriculture. Egypt. J. Biol. Pest Control.

[2] Palma L., Muñoz D., Berry C., Murillo J., Caballero P. (2014). *Bacillus thuringiensis* toxins: An overview of their biocidal activity. Toxins.

[3] Jurat-Fuentes J.L., Heckel D.G., Ferré J. (2021). Mechanisms of resistance to insecticidal proteins from *Bacillus thuringiensis*. Annu. Rev. Entomol..

[4] Chakroun M., Banyuls N., Bel Y., Escriche B., Ferré J. (2016). Bacterial vegetative insecticidal proteins (Vip) from entomopathogenic bacteria. Microbiol. Mol. Biol. Rev..

[5] Chakrabarty S., Jin M., Wu C., Chakraborty P., Xiao Y. (2020). *Bacillus thuringiensis* vegetative insecticidal protein family Vip3A and mode of action against pest Lepidoptera. Pest Manag. Sci..

[6] Syed T., Askari M., Meng Z., Li Y., Abid M.A., Wei Y., Guo S., Liang C., Zhang R. (2020). Current insights on vegetative insecticidal proteins (Vip) as next generation pest killers. Toxins.

[7] Jiang K., Chen Z., Zang Y., Shi Y., Shang C., Jiao X., Cai J., Gao X. (2023). Functional characterization of Vip3Aa from *Bacillus thuringiensis* reveals the contributions of specific domains to its insecticidal activity. J. Biol. Chem..

[8] Jiang K., Zhang Y., Chen Z., Wu D., Cai J., Gao X. (2020). Structural and functional insights into the C-terminal fragment of insecticidal Vip3A toxin of *Bacillus thuringiensis*. Toxins.

[9] Gupta M., Kumar H., Kaur S. (2021). Vegetative insecticidal protein (Vip): A potential contender from *Bacillus thuringiensis* for efficient management of various detrimental agricultural pests. Front. Microbiol..

[10] Ferré J., Bel Y., Lázaro-Berenguer M., Hernández-Martínez P., Jurat-Fuentes J.L. (2023). Chapter Three—Vip3 insecticidal proteins: Structure and mode of action. Advances in Insect Physiology.

[11] Núñez-Ramírez R., Huesa J., Bel Y., Ferré J., Casino P., Arias-Palomo E. (2020). Molecular architecture and activation of the insecticidal protein Vip3Aa from *Bacillus thuringiensis*. Nat. Commun..

[12] Dong F., Zhang S., Shi R., Yi S., Xu F., Liu Z. (2012). Ser-substituted mutations of Cys residues in *Bacillus thuringiensis* Vip3Aa7 exert a negative effect on its insecticidal activity. Curr. Microbiol..

[13] Banyuls N., Hernández-Rodríguez C.S., Van Rie J., Ferré J. (2018). Critical amino acids for the insecticidal activity of Vip3Af from *Bacillus thuringiensis*: Inference on structural aspects. Sci. Rep..

[14] Quan Y., Ferré J. (2019). Structural domains of the *Bacillus thuringiensis* Vip3Af protein unraveled by tryptic digestion of alanine mutants. Toxins.

[15] Banyuls N., Quan Y., González-Martínez R.M., Hernández-Martínez P., Ferré J. (2021). Effect of substitutions of key residues on the stability and the insecticidal activity of Vip3Af from *Bacillus thuringiensis*. J. Invertebr. Pathol..

[16] Yang X., Wang Z., Geng L., Chi B., Liu R., Li H., Gao J., Zhang J. (2022). Vip3Aa domain IV and V mutants confer higher insecticidal activity against *Spodoptera frugiperda* and *Helicoverpa armigera*. Pest Manag. Sci..

[17] Sambrook J., Russel D.W. (2001). Molecular Cloning: A Laboratory Manual.

[18] Şahin B., Gomis-Cebolla J., Günes H., Ferré J. (2018). Characterization of *Bacillus thuringiensis* isolates by their insecticidal activity and their production of Cry and Vip3 proteins. PLoS ONE.

[19] Bell R.A., Joachim F.G. (1976). Techniques for rearing laboratory colonies of tobacco hornworms and pinkboll worms. Ann. Entomol. Soc. Am..

[20] Kahn T.W., Chakroun M., Williams J., Walsh T., James B., Monserrate J., Ferré J. (2018). Efficacy and resistance management potential of a modified Vip3C protein for control of *Spodoptera frugiperda* in maize. Sci. Rep..

[21] Sun Y., Yang P., She M., Lin C., Ye Y., Xu C., Shen Z. (2025). A Vip3Af mutant confers high resistance to broad lepidopteran insect pests. Pest Manag. Sci..

[22] Chi B., Luo G., Zhang J., Sha J., Liu R., Li H., Gao J. (2017). Effect of C-terminus site-directed mutations on the toxicity and sensitivity of *Bacillus thuringiensis* Vip3Aa11 protein against three lepidopteran pests. Biocontrol Sci. Technol..

[23] Chi B., Li H., Zhang J., Wei P., Gao J., Liu R. (2019). In silico structure-based identification and validation of key residues of Vip3Aa involving in lepidopteran brush border receptor binding. Appl. Biochem. Biotechnol..

[24] Beygmoradi A., Homaei A., Hemmati R., Fernandes P. (2023). Recombinant protein expression: Challenges in production and folding related matters. Int. J. Biol. Macromol..

[25] Rong Y., Jensen S.I., Lindorff-Larsen K., Nielsen A.T. (2023). Folding of heterologous proteins in bacterial cell factories: Cellular mechanisms and engineering strategies. Biotechnol. Adv..

[26] Francis D.M., Page R. (2010). Strategies to optimize protein expression in *E. Coli*. Curr. Protoc. Protein Sci..

