# Supplementary material for: Amino Acid Substitutions in the C-Terminal Domains of Vip3Aa Enhance Insecticidal Toxicity
Source: Biology (Basel). 2026 Sep 3;15(17):1508. doi: 10.3390/biology15171508 (PMC13565636; doi:10.3390/biology15171508)
Supplement: Supplementary file 1 [file biology-15-01508-s001.zip › biology-4491945-supplementary.pdf]

Supplementary Material for:

Amino acid substitutions in the C-terminal domains of Vip3Aa enhance insecticidal toxicity

Burcu Şahin [1], Patricia Hernández-Martínez [2] and Juan Ferré [2,*]

[1] Department of Biology, Faculty of Science, Muğla Sıtkı Koçman University, 48000, Muğla, Türkiye
[2] Institute of Biotechnology and Biomedicine (BIOTECMED), Department of Genetics, Universitat de València, 46100 Burjassot, Spain
[*] Correspondence: juan.ferre@uv.es; Tel.: (34) 96 354 4506

Supplementary Figure S1

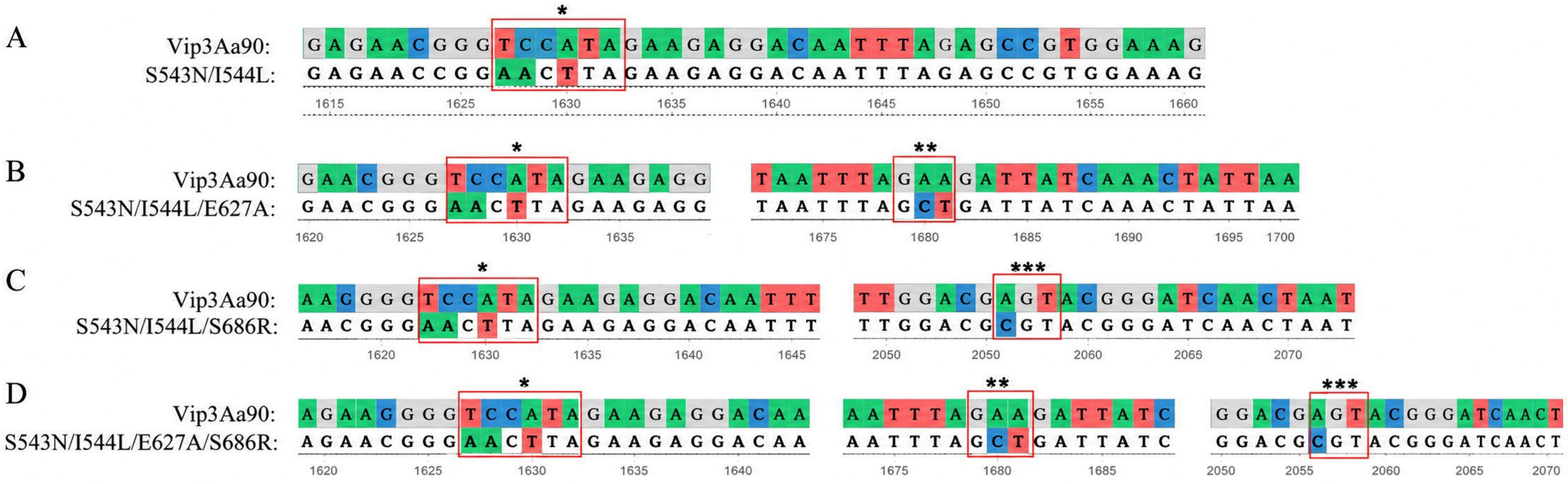

Figure S1. Sequence analysis of mutant Vip3Aa90 constructs. The mutation sites are shown in the red boxes.

*: S543N/I544L mutant plasmid carries a TCC-to-AAC substitution at nucleotides 1627–1629 and a ATA-to-TTA substitution at nucleotides 1630-1632.
**: E627A mutant plasmid carries a GAA-to-GCT substitution at nucleotides 1879-1881.
***: S686R mutant plasmid carries a AGT-to-CGT substitution at nucleotides 2056-2058.

Supplementary Figure S2

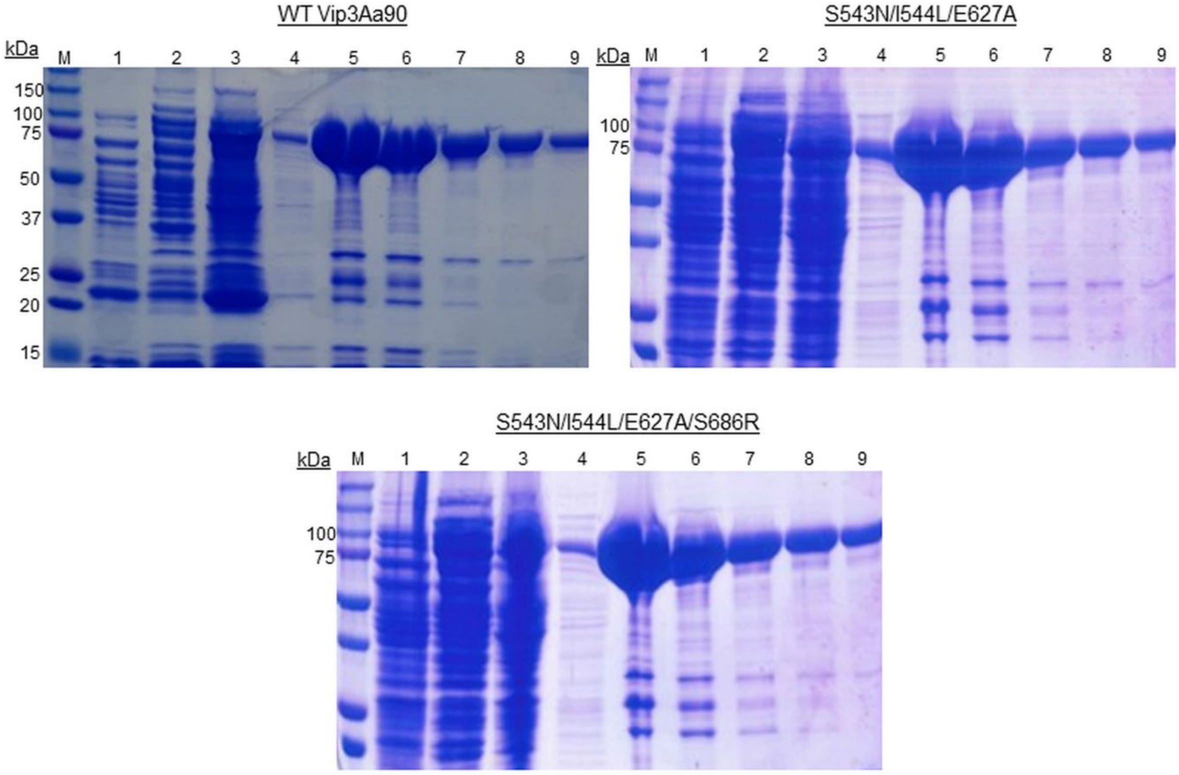

Figure S2. Purification of WT and mutant S543N/I544L/E627A and S543N/I544L/E627A/S686R Vip3Aa90 proteins by affinity chromatography using His-Trap FF columns. M: Molecular Weight Marker, 1: lysate, 2: flow through, 3: washing, 4: first elution fraction, 5-9: elution fractions to be combined for assays.

Supplementary Figure S3

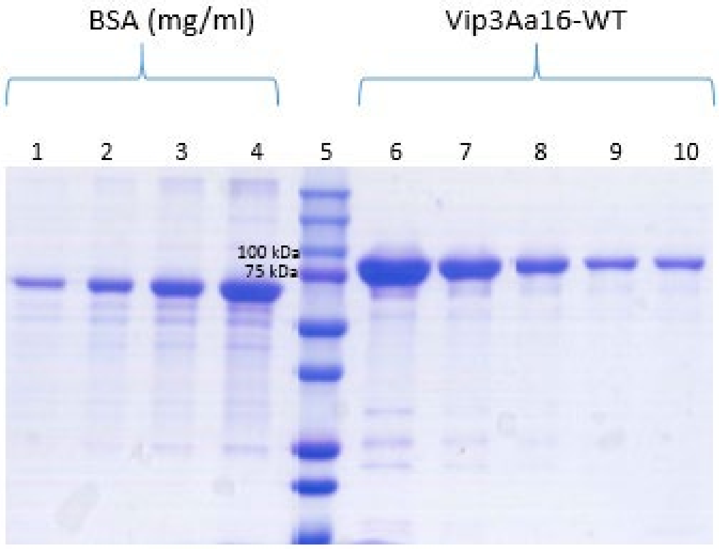

Figure S3. SDS-PAGE gel for densitometry analysis of WT Vip3Aa90 protein. 1: BSA (0.4 mg/ml), 2: BSA (0.2 mg/ml), 3: BSA (0.1 mg/ml), 4: BSA (0.05 mg/ml), 5: Molecular Weight Marker, 6: 1/2 dilution of elution fractions (ef) 1 to 5, 7: 1/4 dilution of ef 1 to 5, 8: 1/8 dilution of ef 1 to 5, 9: 1/16 dilution of ef 1 to 5, 10: ef 6 to 7.

Supplementary Figure S4

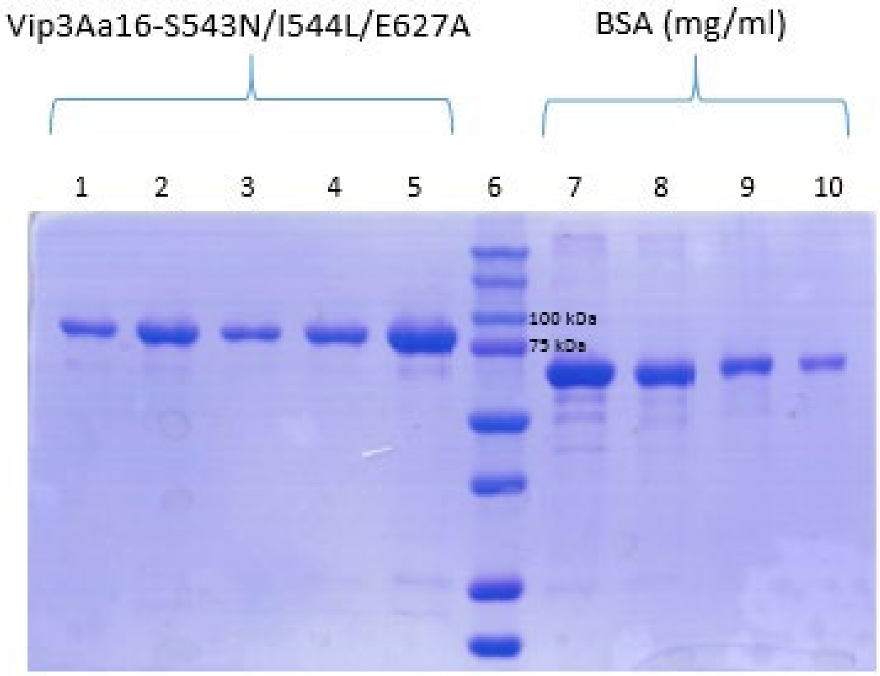

Figure S4. SDS-PAGE gel for densitometry analysis of Vip3Aa90- S543N/I544L/E627A protein. 1: 1/16 dilution of ef 2 to 3, 2: 1/8 dilution of ef 2 to 3, 3: 1/64 dilution of ef1, 4: 1/32 dilution of ef1, 5: 1/16 dilution of ef1, 6: Molecular Weight Marker, 7: BSA (0.4 mg/ml), 8: BSA (0.2 mg/ml), 9: BSA (0.1 mg/ml), 10: BSA (0.05 mg/ml)

Supplementary Figure S5

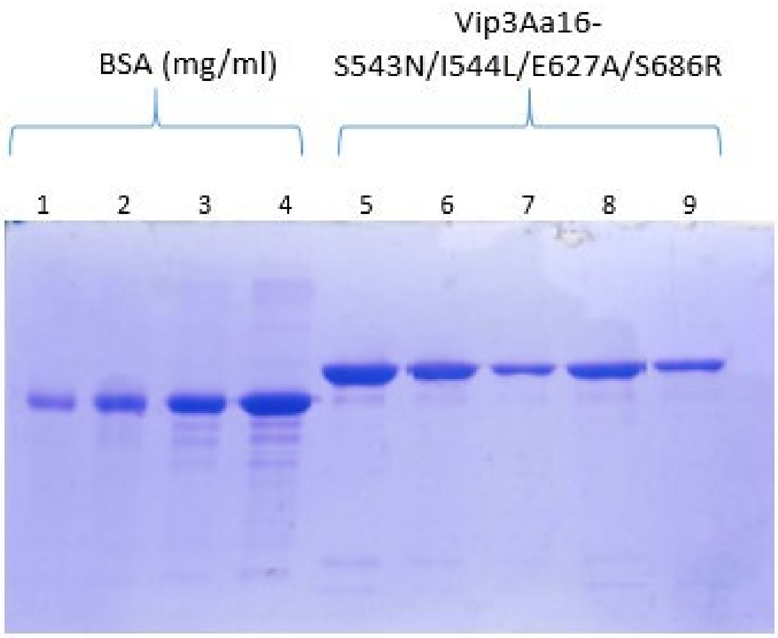

Figure S5. SDS-PAGE gel for densitometry analysis of Vip3Aa90-S543N/I544L/E627A/S686R protein. 1: BSA (0.05 mg/ml), 2: BSA (0.1 mg/ml), 3: BSA (0.2 mg/ml), 4: BSA (0.4 mg/ml), 5: 1/16 dilution of ef1, 6: 1/32 dilution of EF1, 7: 1/64 dilution of EF1, 8: 1/8 dilution of ef 2 to 3, 9: 1/16 dilution of ef 2 to 3.

Supplementary Table S1. The concentrations of Vip3Aa90 WT and mutant protoxins and numbers of dead larvae for each replicate against *S. littoralis*

**Vip3Aa90 protoxin**	**WT**			**S543N/I544L/E627A**			**S543N/I544L/E627A/S686R**		
**Protein concentration (ng/cm^2)**	**Replicate 1**	**Replicate 2**	**Replicate 3**	**Replicate 1**	**Replicate 2**	**Replicate 3**	**Replicate 1**	**Replicate 2**	**Replicate 3**
180	16	14							
60	12	16	16	16	16		16	16	16
20	11	8	14	15	15	16	16	15	16
6.6	6	7	3	13	10	12	13	9	13
2.2	3		4	6	4	2	7	5	4
0.73	2	3	2		3	2	4	1	0
0.24		5	3						0
Negative control	1	2	0	0	3	0	1	1	3

Supplementary Table S2. The concentrations of Vip3Aa90 WT and mutant protoxins and numbers of dead larvae for each replicate against *S. exigua*

Vip3Aa90 protoxin	WT			S543N/I544L/E627A		S543N/I544L/E627A/S686R		
Protein concentration (ng/cm^2)	Replicate 1	Replicate 2	Replicate 3	Replicate 1	Replicate 2	Replicate 1	Replicate 2	Replicate 3
85	16	16	15			16	16	16
28.3	15	16	7	16	16	16	15	15
9.4				16	14	9	5	6
3.1	6	12	2	10	10	3	2	3
1.03	5	5	2	2	5	1	0	0
0.34	3	6	1	2	2			
0.11				1				
Negative control	1	1	1	2	0	0	1	1

Supplementary Table S3. The concentrations of Vip3Aa90 WT and mutant protoxins and numbers of dead larvae for each replicate against *G. molesta*

**Vip3Aa90 protoxin**	**WT**		**S543N/I544L/E627A**			**S543N/I544L/E627A/S686R**		
**Protein concentration (ng/cm^2)**	**Replicate 1**	**Replicate 2**	**Replicate 1**	**Replicate 2**	**Replicate 3**	**Replicate 1**	**Replicate 2**	**Replicate 3**
405						13	16	11
135		15	16	15	13	9	16	5
45	16	12	16	12	14	4	10	2
15	10	7	11	4	11	1	7	2
5	3	6	10	2	5	0	5	0
1.6	2	1	4	2	4			
Negative control	0	0	1	1	3	0	0	0
